# Supplementary figures and images for: Berberine inhibits free fatty acid and LPS-induced inflammation via modulating ER stress response in macrophages and hepatocytes
Source: PLoS One. 2020 May 1;15(5):e0232630. doi: 10.1371/journal.pone.0232630 (PMC7194368; doi:10.1371/journal.pone.0232630)

Supplementary Fig. S1.

A

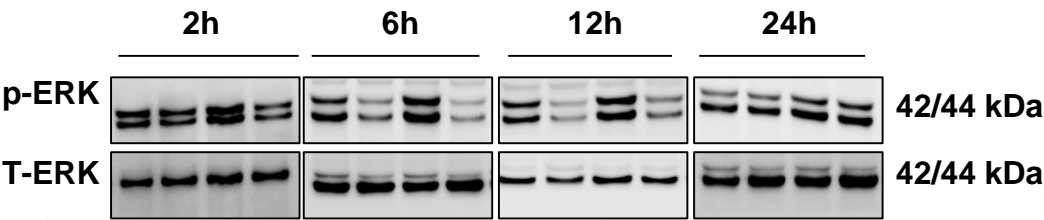

B

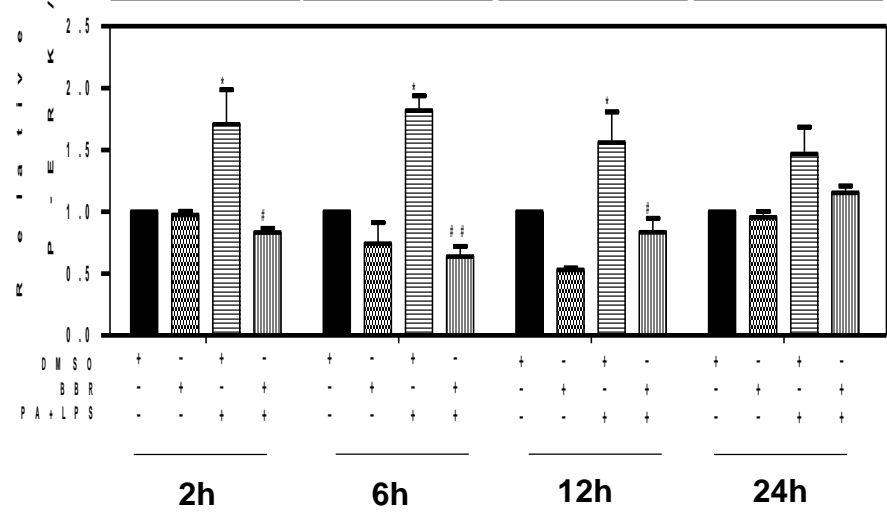

Supplement: S1 Fig — RAW264.7 cells were pre-treated with BBR (5 μM) for 1 h, then treated with PA(0.25mM) and LPS (25 ng/mL) for 2, 6, 12 and 24h. Total cell lysates were prepared for Western blot analysis as described under Materials and Methods. Values are mean ± S.E. of three independent experiments. Statistical significance relative to vehicle control, *p<0.05; relative to PA+LPS, #p<0.05, ##p<0.01. A. Representative immunoblots of phospho(p)-ERK and total (T)-ERK; B. The relative protein levels of p-ERK. (PDF) [file pone.0232630.s001.pdf]

### Supplementary Fig.S2.

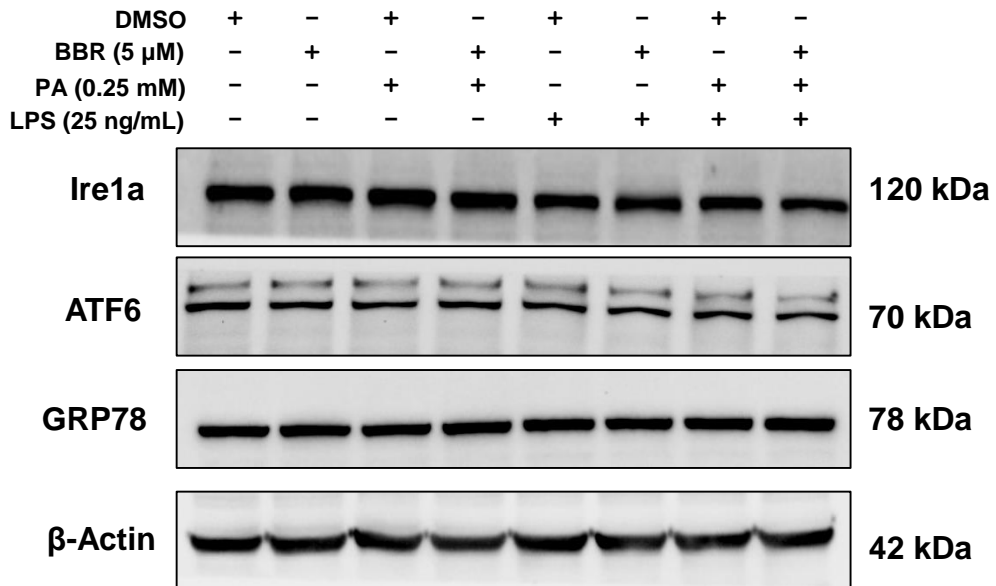

Supplement: S2 Fig — RAW264.7 cells are pre-treated with BBR (5 μM) for 1 h, then treated with PA (0.25 mM) and LPS (25 ng/mL) for 6 h. Total cell lysates were prepared. The protein expression levels of IRE1α, ATF6, GRP78, and β-Actin were measured by Western blot analysis as described under Materials and Methods. β-Actin was used as the loading control. A. Representative immunoblots of IRE1α, ATF6, GRP78, and β-Actin are shown. (PDF) [file pone.0232630.s002.pdf]

Supplementary Fig.S3

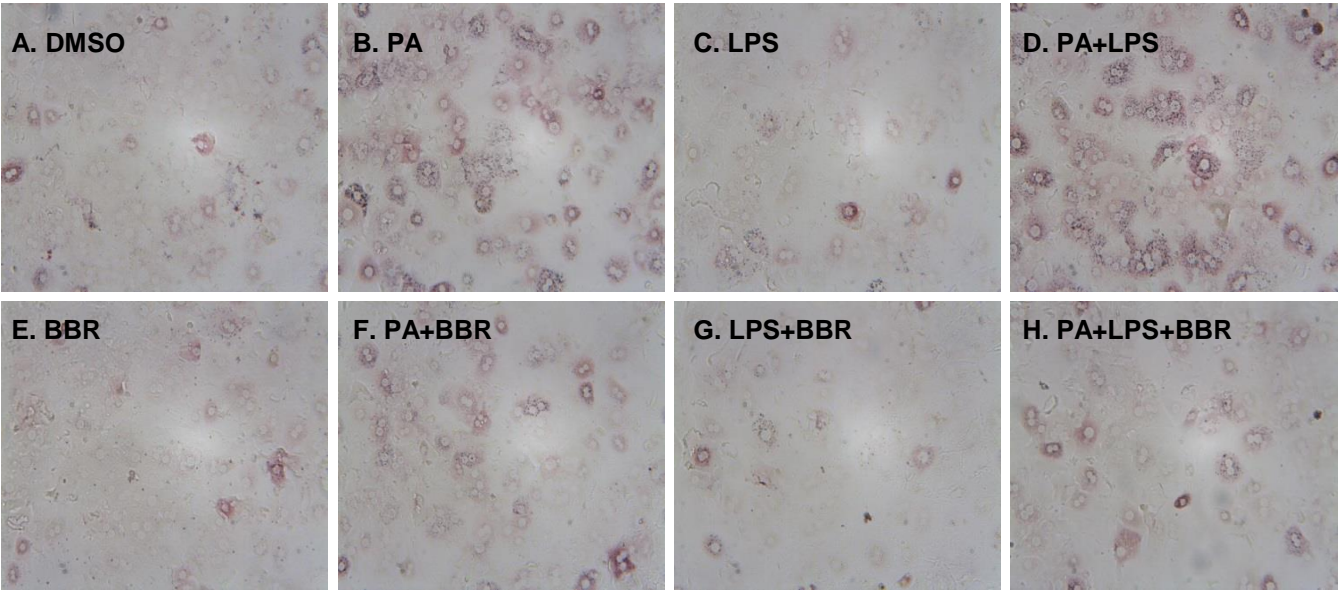

Supplement: S3 Fig — Primary mouse hepatocytes were plated on 22 × 22-mm glass coverslips in 6-well plates. Hepatocytes were pre-treated with BBR (5 μM) for 1 h, then treated with PA (0.25 mM) or LPS (25 ng/mL) or both for 6 h. At the end of the treatment, hepatocytes were fixed with 3.7% formaldehyde in PBS for 30 min followed by two washes with PBS. The hepatocytes were stained with 0.2% Oil Red O in 60% 2-propanol for 10 min and washed three times with PBS. The images of Oil Red O staining were taken with a microscope (Olympus, Tokyo, Japan) equipped with an image recorder under a 10 × lens. A. DMSO; B. BBR; C. PA; D.PA+BBR; E. LPS; F.LPS+BBR; G.PA/LPS; H. PA/LPS+BBR. (PDF) [file pone.0232630.s003.pdf]
