# Supplementary material for: Berberine inhibits free fatty acid and LPS-induced inflammation via modulating ER stress response in macrophages and hepatocytes
Source: PLoS One. 2020 May 1;15(5):e0232630. doi: 10.1371/journal.pone.0232630 (PMC7194368; doi:10.1371/journal.pone.0232630)
Supplement: S1 Table — (DOCX) [file pone.0232630.s004.docx]

Supplementary Table 1.

List of antibodies

| **Antibody** | **Species** | **Source** | **Catalog #** | **Dilution** |
| --- | --- | --- | --- | --- |
| CHOP | Mouse | Santa Cruz | sc-7351 | 1:500 |
| ATF4 | Mouse | Santa Cruz | sc-390063 | 1:500 |
| XBP-1 | Mouse | Santa Cruz | sc-8015 | 1:500 |
| p-ERK | Mouse | Santa Cruz | sc-7383 | 1:500 |
| ERK1 | Mouse | Santa Cruz | sc-271269 | 1:500 |
| ERK2 | Mouse | Santa Cruz | sc-1647 | 1:500 |
| IL-1β | Mouse | Santa Cruz | sc-515598 | 1:500 |
| GRP78 | Rabbit | Cell Signaling | 3177S | 1:1000 |
| IRE1α | Rabbit | Santa Cruz | sc-20790 | 1:500 |
| β-Actin (JLA20) | Mouse | DSHB University of Iowa | JLA20 | 1:500 |
