# Supplementary material for: Berberine inhibits free fatty acid and LPS-induced inflammation via modulating ER stress response in macrophages and hepatocytes
Source: PLoS One. 2020 May 1;15(5):e0232630. doi: 10.1371/journal.pone.0232630 (PMC7194368; doi:10.1371/journal.pone.0232630)
Supplement: S2 Table — (DOCX) [file pone.0232630.s005.docx]

Supplementary Table 2.

List of QPCR primers

| Gene | Forward | Reverse |
| --- | --- | --- |
| HPRT1 | 5′ CAG ACT TTG TTG GAT TTG AAA 3′ | 5′ GCT CAT CTT AGG CTT GTA T 3′ |
| TNF-α | 5′ GGTGCCTATGTCTCAGCCTCTT 3′ | 5′ GCCATAGAACTGATGAGAGGGAG 3′ |
| IL-6 | 5′ GAG GAT ACC ACT CCC AAC AGA CC 3′ | 5′ AAG TGC ATC ATC GTT GTT CAT ACA 3′ |
| IL-1β | 5′ AAT CTC ACA GCA GCA CAT C 3′ | 5′ AGC AGG TTA TCA TCA TCA TCC 3′ |
| MCP-1 | 5’ GCTACAAGAGGATCACCAGCAG 3’ | 5’ GTCTGGACCCATTCCTTCTTGG 3’ |
| CHOP | 5′ GTC CCT GCC TTT CAC CTT GG 3′ | 5′ GGT TTT TGA TTC TTC CTC TTC G 3′ |
| ATF4 | 5′ CCT AGG TCT CTT AGA TGA CTA TCT GGA GG 3′ | 5′ CCA GGT CAT CCA TTC GAA ACA GAG CAT CG 3′ |
| XBP-1u | 5′ CGC AGC ACT CAG ACT ATG 3′ | 5′ TTC CTC CAG ACT AGC AGA C 3′ |
| XBP-1s | 5′ TGA GTC CGC AGC AGG TG 3′ | 5′ GAC AGG GTC CAA CTT GT 3′ |
