# Supplementary material for: Berberine inhibits free fatty acid and LPS-induced inflammation via modulating ER stress response in macrophages and hepatocytes
Source: PLoS One. 2020 May 1;15(5):e0232630. doi: 10.1371/journal.pone.0232630 (PMC7194368; doi:10.1371/journal.pone.0232630)
Supplement: S1 Raw image — (PDF) [file pone.0232630.s006.pdf]

a. (Fig. 2D)

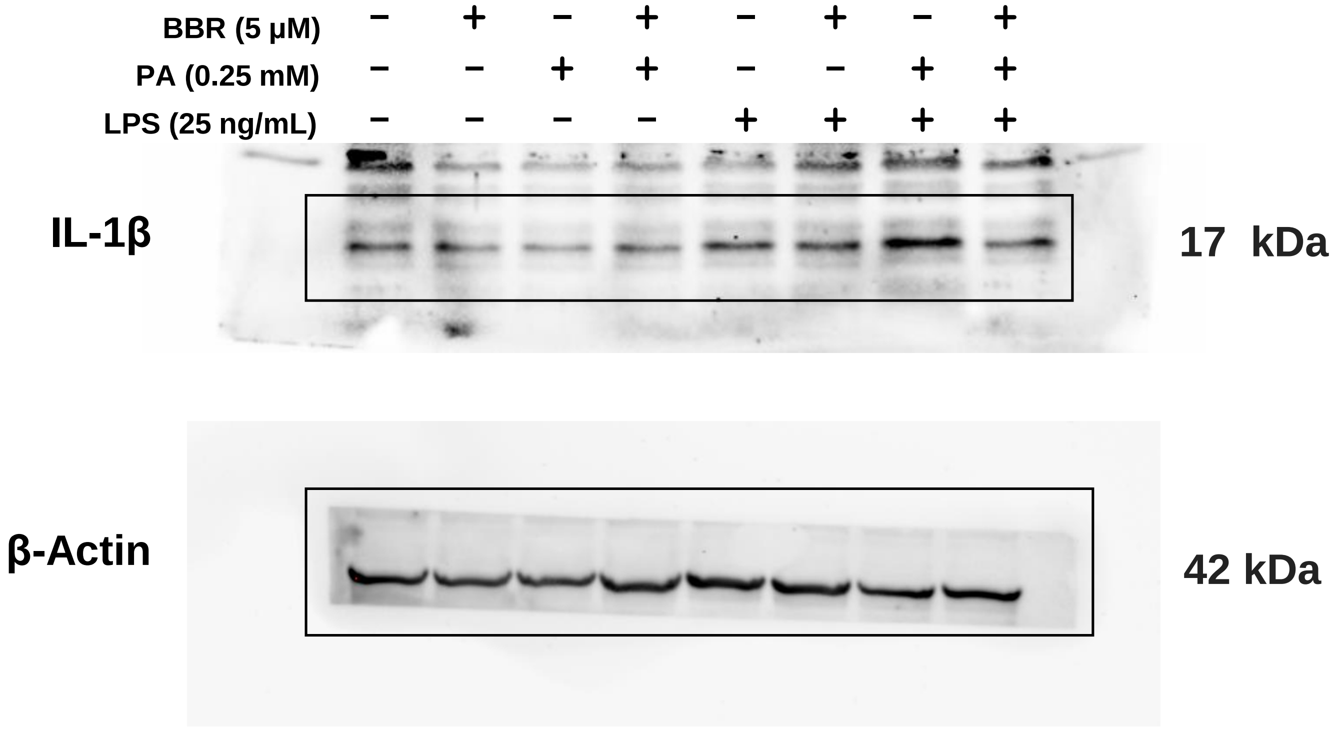

Captured by ChemiDoc MP Imaging System

b. (Fig. 4A)

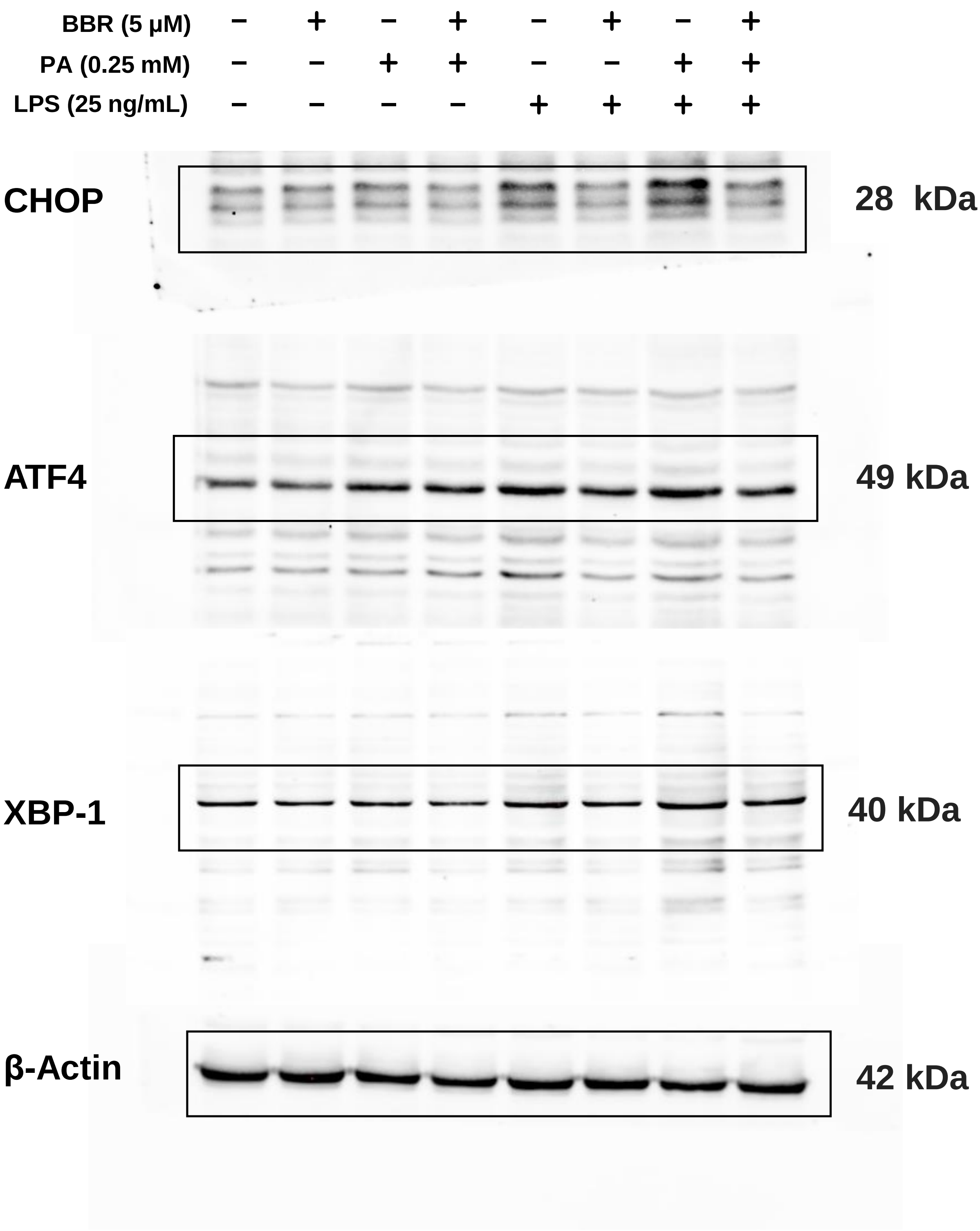

Captured by ChemiDoc MP Imaging System

c. (Fig. 5)

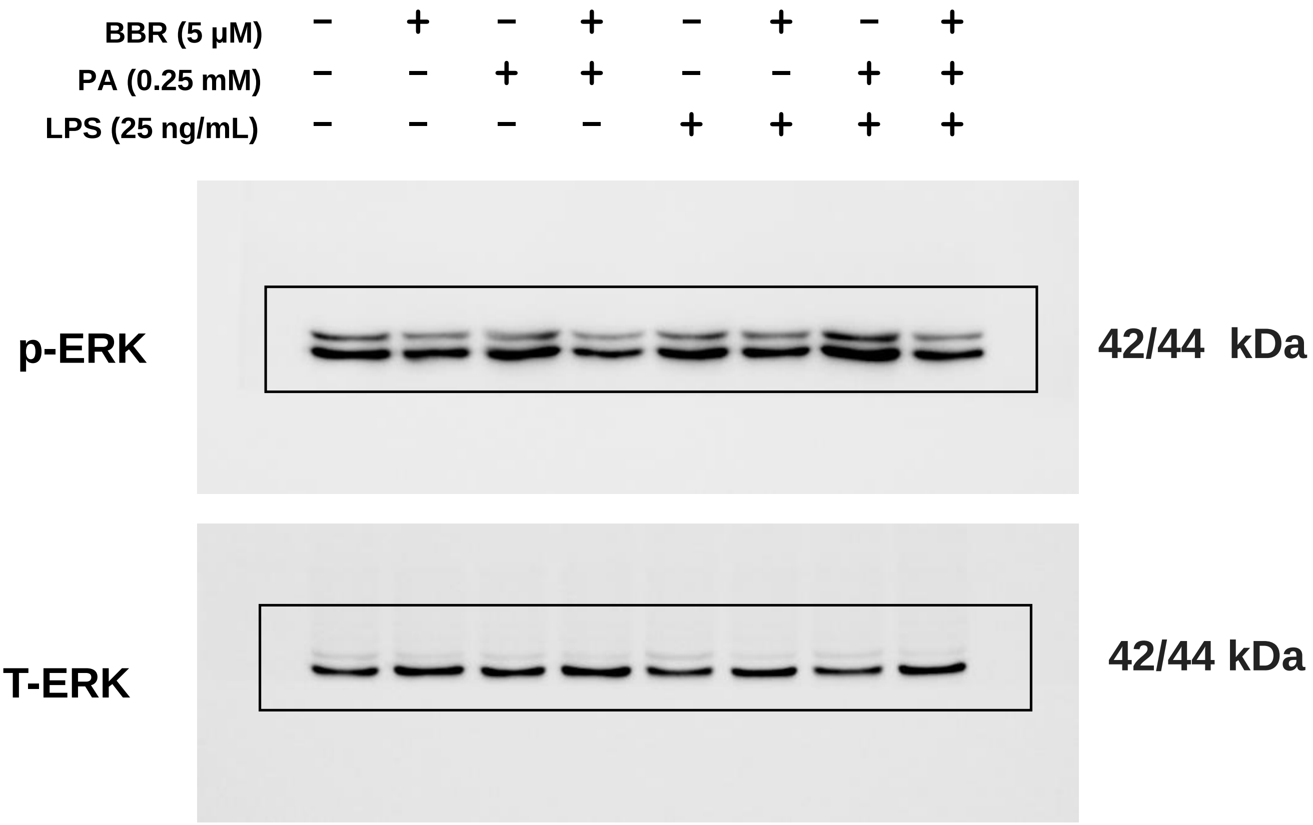

Captured by ChemiDoc MP Imaging System

d. (Fig. 6)

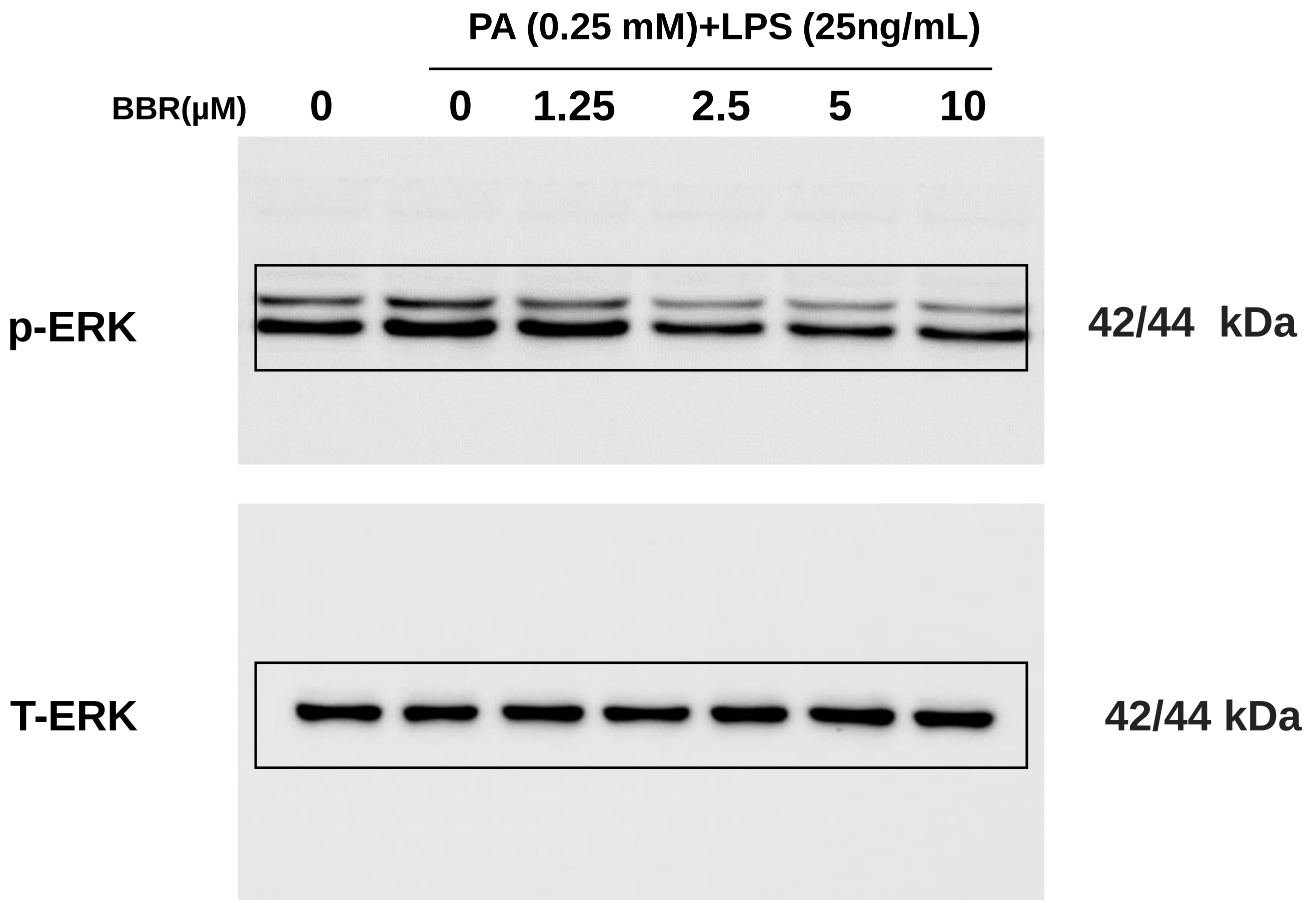

Captured by ChemiDoc MP Imaging System

e. (Fig. 7B)

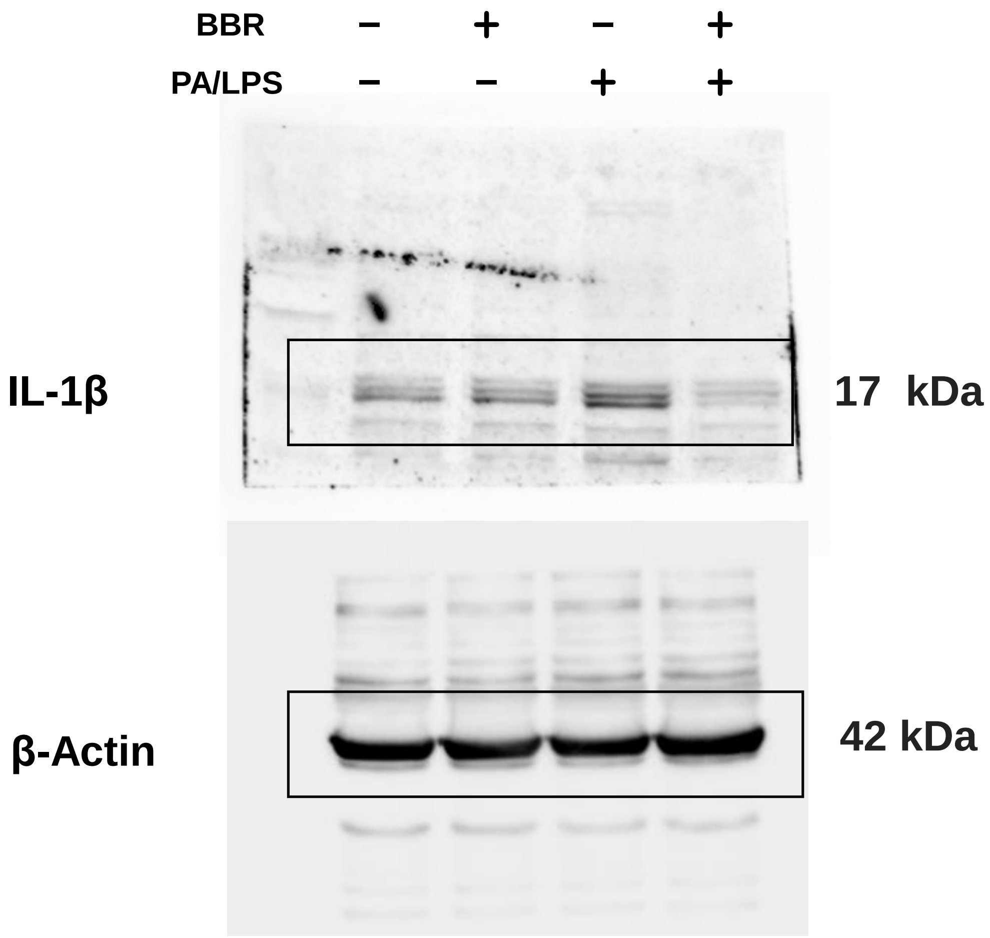

Captured by ChemiDoc MP Imaging System

f. (Fig. 7D)

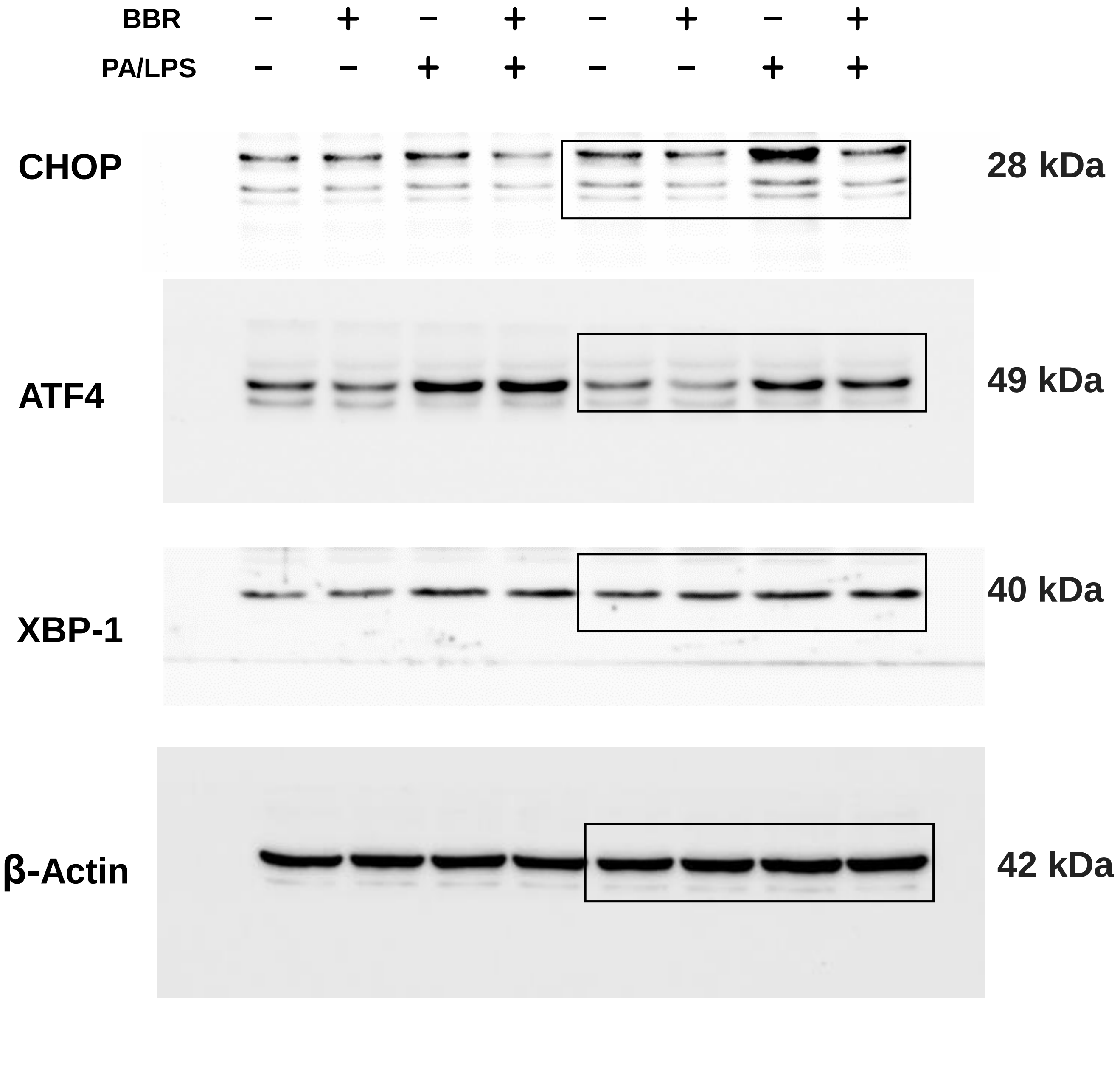

Captured by ChemiDoc MP Imaging System

g. (Fig. 7F)

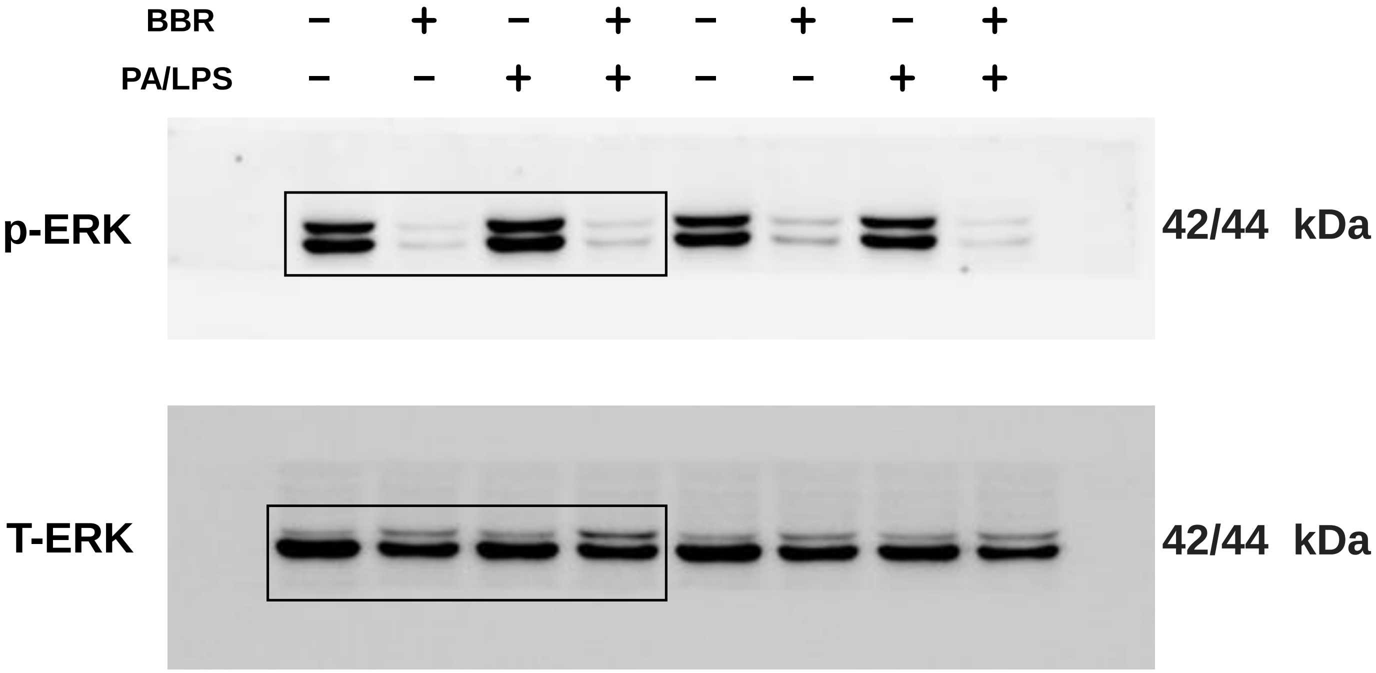

Captured by ChemiDoc MP Imaging System

h. (Fig. S1)

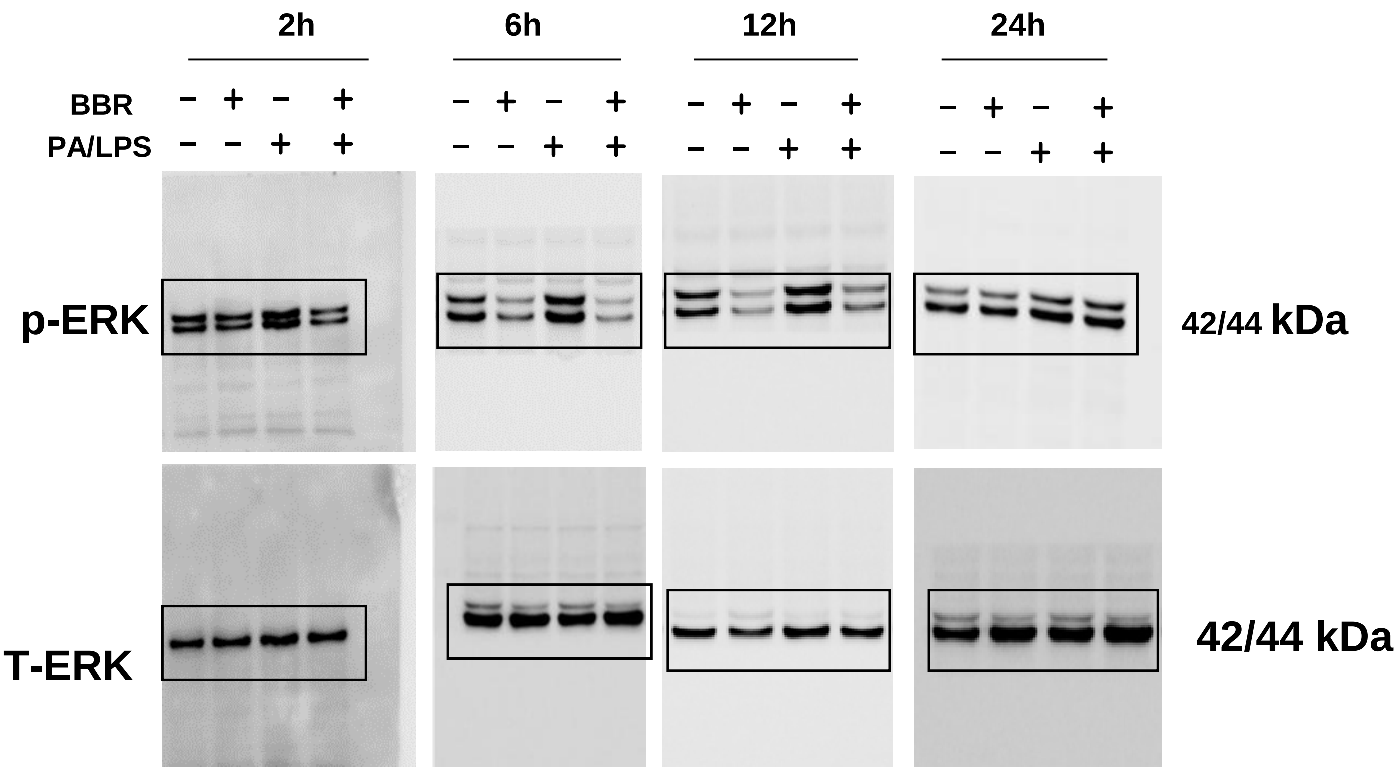

Captured by ChemiDoc MP Imaging System

i. (Fig. S2)

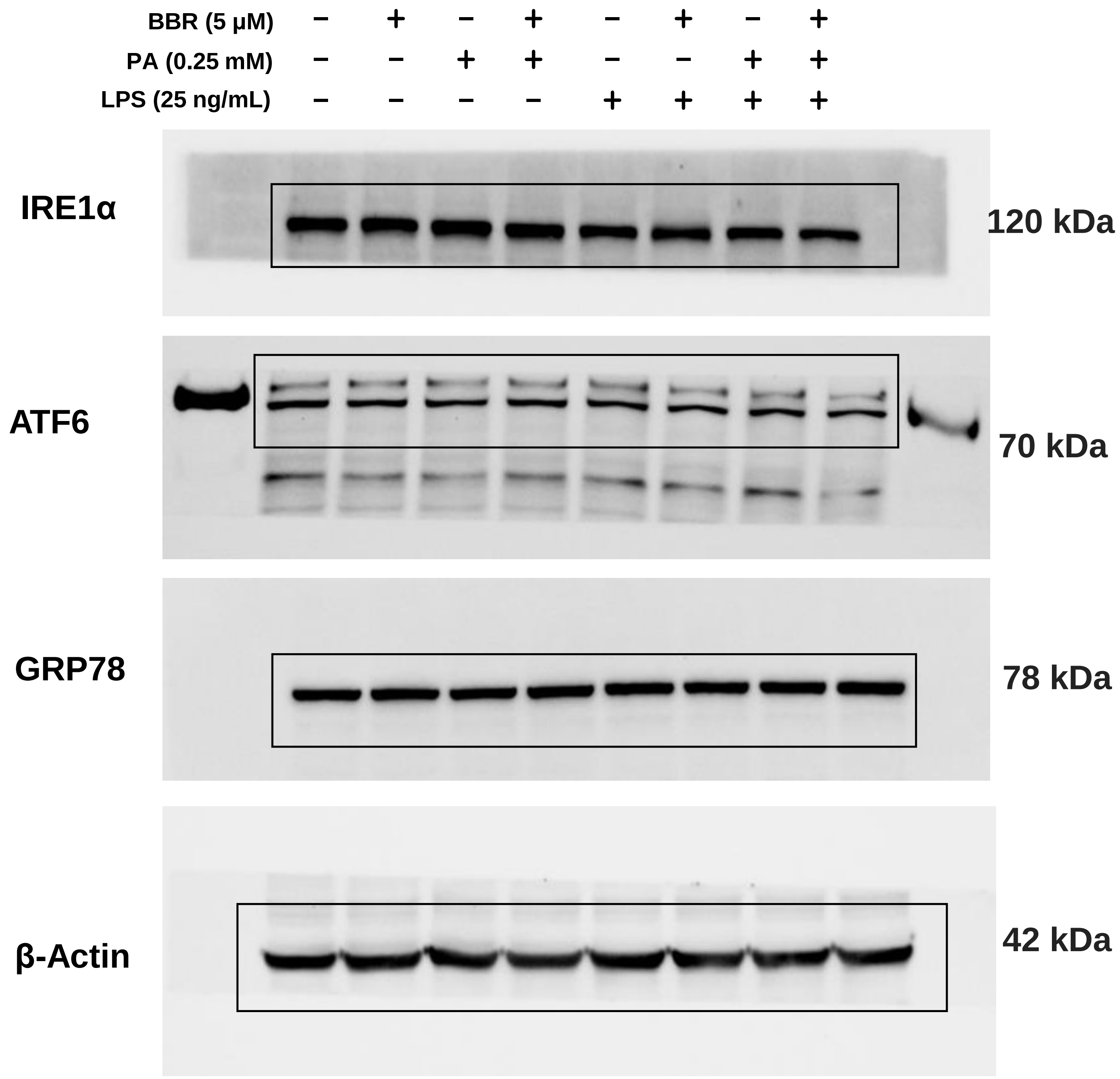

Captured by ChemiDoc MP Imaging System
