# Supplementary material for: Berberine inhibits free fatty acid and LPS-induced inflammation via modulating ER stress response in macrophages and hepatocytes
Source: PLoS One. 2020 May 1;15(5):e0232630. doi: 10.1371/journal.pone.0232630 (PMC7194368; doi:10.1371/journal.pone.0232630)
Supplement: Attachment — Submitted filename: Response to reviewer-Final-R.docx [file pone.0232630.s007.docx]

**Response to Reviewer’s comment**

**Comment:** A minor recommendation is to further provide the known mechanisms of action of BBR in the context of NAFLD and NASH studies, and how this study provides in vitro evidence supporting or contracting the known effects of BBR on liver lipid metabolism and inflammation.

**Response:** We would like to thank the Reviewer for his or/her time spent reviewing our manuscript and helpful comment. In the revised manuscript, we provided the information regarding the potential mechanisms of Berberine-mediated therapeutic effect on NAFLD/NASH based on the most recent studies in the discussion.
